# Leveraging Large Language Models for Improved Understanding of Communications With Patients With Cancer in a Call Center Setting: Proof-of-Concept Study

**DOI:** 10.2196/63892

**Published:** 2024-12-11

**Authors:** Seungbeom Cho, Mangyeong Lee, Jaewook Yu, Junghee Yoon, Jae-Boong Choi, Kyu-Hwan Jung, Juhee Cho

**Affiliations:** 1 School of Mechanical Engineering Sungkyunkwan University Suwon-si Republic of Korea; 2 Department of Digital Health, Samsung Advanced Institute for Health Sciences and Technology Sungkyunkwan University Seoul Republic of Korea; 3 Center for Clinical Epidemiology Samsung Medical Center Seoul Republic of Korea; 4 Department of Clinical Research Design and Evaluation, Samsung Advanced Institute for Health Sciences and Technology Sungkyunkwan University Seoul Republic of Korea; 5 Service Convergence Design (Interdisciplinary) Sungkyunkwan University Suwon-si Republic of Korea; 6 Department of Medical Device Management and Research, Samsung Advanced Institute for Health Sciences and Technology Sungkyunkwan University Seoul Republic of Korea; 7 Smart Healthcare Research Center, Research Institute for Future Medicine Samsung Medical Center Seoul Republic of Korea; 8 Cancer Education Center Samsung Comprehensive Cancer Center Seoul Republic of Korea

**Keywords:** large language model, cancer, supportive care, LLMs, patient communication, natural language processing, NLP, self-management, teleconsultation, triage services, telephone consultations

## Abstract

**Background:**

Hospital call centers play a critical role in providing support and information to patients with cancer, making it crucial to effectively identify and understand patient intent during consultations. However, operational efficiency and standardization of telephone consultations, particularly when categorizing diverse patient inquiries, remain significant challenges. While traditional deep learning models like long short-term memory (LSTM) and bidirectional encoder representations from transformers (BERT) have been used to address these issues, they heavily depend on annotated datasets, which are labor-intensive and time-consuming to generate. Large language models (LLMs) like GPT-4, with their in-context learning capabilities, offer a promising alternative for classifying patient intent without requiring extensive retraining.

**Objective:**

This study evaluates the performance of GPT-4 in classifying the purpose of telephone consultations of patients with cancer. In addition, it compares the performance of GPT-4 to that of discriminative models, such as LSTM and BERT, with a particular focus on their ability to manage ambiguous and complex queries.

**Methods:**

We used a dataset of 430,355 sentences from telephone consultations with patients with cancer between 2016 and 2020. LSTM and BERT models were trained on 300,000 sentences using supervised learning, while GPT-4 was applied using zero-shot and few-shot approaches without explicit retraining. The accuracy of each model was compared using 1,000 randomly selected sentences from 2020 onward, with special attention paid to how each model handled ambiguous or uncertain queries.

**Results:**

GPT-4, which uses only a few examples (a few shots), attained a remarkable accuracy of 85.2%, considerably outperforming the LSTM and BERT models, which achieved accuracies of 73.7% and 71.3%, respectively. Notably, categories such as “Treatment,” “Rescheduling,” and “Symptoms” involve multiple contexts and exhibit significant complexity. GPT-4 demonstrated more than 15% superior performance in handling ambiguous queries in these categories. In addition, GPT-4 excelled in categories like “Records” and “Routine,” where contextual clues were clear, outperforming the discriminative models. These findings emphasize the potential of LLMs, particularly GPT-4, for interpreting complicated patient interactions during cancer-related telephone consultations.

**Conclusions:**

This study shows the potential of GPT-4 to significantly improve the classification of patient intent in cancer-related telephone oncological consultations. GPT-4’s ability to handle complex and ambiguous queries without extensive retraining provides a substantial advantage over discriminative models like LSTM and BERT. While GPT-4 demonstrates strong performance in various areas, further refinement of prompt design and category definitions is necessary to fully leverage its capabilities in practical health care applications. Future research will explore the integration of LLMs like GPT-4 into hybrid systems that combine human oversight with artificial intelligence–driven technologies.

## Introduction

Patients with cancer face various physical, psychosocial, and financial problems throughout their diagnosis, treatment, and recovery [1-4]. These multifactorial problems often increase patient distress and make it difficult for patients to commit themselves to their treatment [5]. Missed opportunities for timely intervention can lead to an impaired quality of life, reduced treatment effectiveness, poor prognosis, and even death [6-9]. Therefore, timely and accurate identification of problems raised by patients is important [10].

Operating a call center in a hospital is an effective way for patients to access information and support to ensure quality care. One-third of patients receive at least one telephone consultation [11,12]. In addition to informational support, patients can also advance to scheduled clinical visits due to worsening conditions and receive brief instructions for self-management. Moreover, one study reported that incorporating nurse practitioners into telephone triage services was associated with 30% fewer subsequent health care encounters [13].

Despite the clinical contributions of telephone consultations, there are still challenges relating to their operational efficiency and standardization. Patient satisfaction with call centers is associated with the average speed of answers [14]. In addition, poor knowledge among nurses in identifying and prioritizing patients’ problems leads to patient dissatisfaction and slows clinical workflows [15-17]. In another study with nurses, 97% felt constant pressure to minimize call length, but only 27% agreed that there was standardized guidance supporting clinical decisions during calls [18]. Given that most call center workers are not medical professionals, these challenges may be held.

To address these concerns, there is growing interest in leveraging natural language processing, particularly to understand patient intents [19]. Because most existing consultation records lack data with accurate annotations about patient intents, fine-tuning of the pretrained language model is required. There have been well-documented studies on the performance of pretrained language model, such as bidirectional encoder representations from transformers (BERT), in clinical contexts [20,21]. However, this approach is slightly limited in that it may still depend on the quality and quantity of annotation, which is time-consuming and costly to acquire in real-world clinical settings. In contrast, large language models (LLMs), such as the GPT-3, which leverages billions of words and parameters, are known to better understand contextual information without explicit fine-tuning by leveraging in-context learning capabilities [22-24]. Therefore, this study evaluated whether LLMs can effectively understand complex and diverse queries during telephone consultations with patients with cancer. We also assessed the impact of uncertainty in the annotations of consultation data on the performance of the LLM models, with a particular focus on how such uncertainties affect model accuracy.

## Methods

### Overall Process

**Figure 1:**
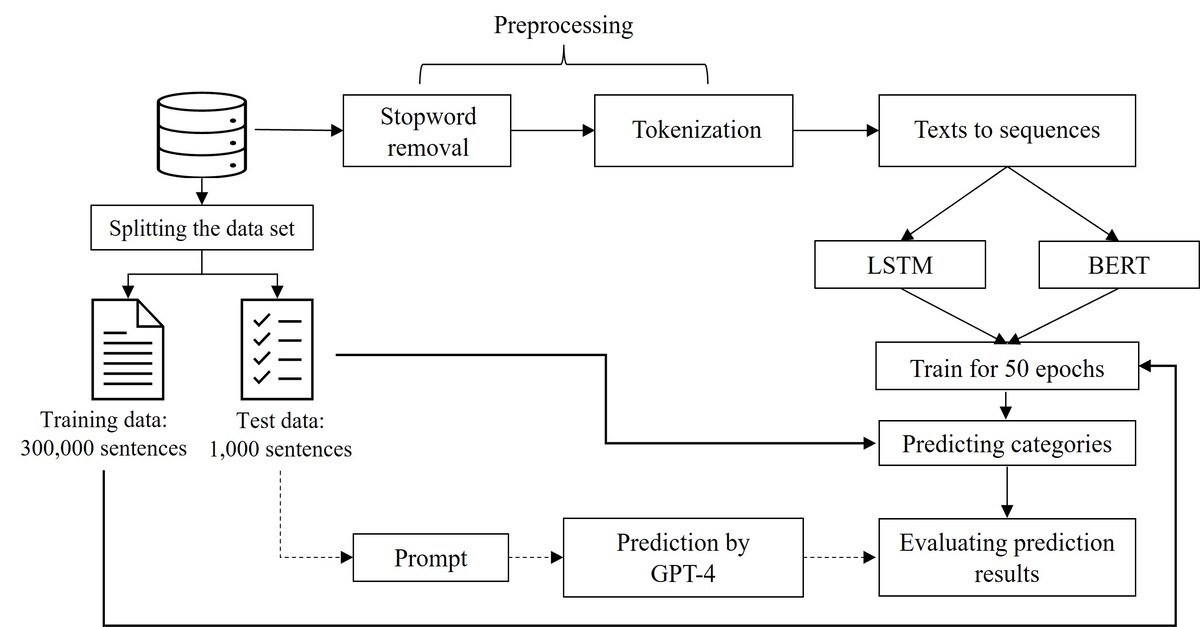
Process of applying discriminative deep learning models for classifying the purpose of telephone consultations with patients with cancer. BERT: bidirectional encoder representations from transformers; LSTM: long short-term memory.

The overall process of applying discriminative deep learning models and generative LLM to classification tasks is presented in Figure 1. The purpose of phone calls is based on the transcripts of calls of patients with cancer as described. Initially, phone call transcripts were processed by converting them into a corpus through tokenization postremoval of stop words. Subsequently, each text segment within the transcripts underwent conversion into token sequences. These sequences were used to train and test discriminant deep learning models such as long short-term memory (LSTM) and BERT using supervised learning.

Despite the effectiveness of supervised deep learning models in discriminative tasks when a high-quality annotated dataset is available, the subjective and uncertain characteristics of phone call purpose categorization can lead to degraded performance. To address this issue, this study explored the potential of using GPT-4, a chatbot powered by a generative LLM to categorize phone calls without explicitly training the model with annotated data. Finally, we compared the performances of the two approaches with respect to the categorization accuracy.

### Data Preparation

#### Datasets

This study used real-world data collected between 2016 and 2020 from the customer service center of a university hospital in Seoul, Korea. Specifically, we analyzed 430,355 instances documenting the initial requirements of patients when they contacted the hospital. All incoming calls were initially handled by the customer service center, after which counselors redirected them to the appropriate departments based on patients’ unmet needs, categorized into 17 prespecified call purposes. Counselors are typically trained to write the exact words of patients verbatim when hearing their unmet needs, ensuring clear communication and understanding when the information is passed on to other departments from the customer service center. The records were all documented in Korean.

Approximately 300,000 of these sentences from data up to 2019 were used as training data, whereas 1,000 randomly selected sentences from the remaining 2020 data were used as the test dataset. The training dataset for the deep learning model was divided at an 80:20 ratio, with 20% used for validation during the training phase. The test data were categorized into 2 types: “Initial Categories,” classified by counselors in the customer service center, and “Refined Categories,” redefined by an oncology nurse and two researchers experienced in educating cancer patients or supportive care study for patients with cancer.

#### Definition and Refinement of Categories

The “Treatment” category encompasses consultations related to diagnosis, surgery, procedures, medication, dental treatment, and includes both therapeutic and procedural aspects of patient care. “Records” focuses on inquiries related to medical documents, including access to, interpretation of, and correction of medical records and opinions. The “Routine” category includes vaccination advice, such as flu shots, as well as lifestyle questions that emphasize the value of preventative care and routine health care. “Rescheduling” encompasses all aspects of organizing and coordinating medical appointments, surgeries, and treatments. The “Symptom” category includes issues related to medication side effects and symptoms occurring after an illness or treatment. A detailed description of each category is provided in Table S1 in Multimedia Appendix 1.

### Model Development and Training

#### Training of the Discriminative Deep Learning Models

Two popular models widely used for processing sequence data in deep learning, LSTM, and BERT, were applied for classification. LSTMs, which are variants of recurrent neural networks (RNNs), are structurally more complex than RNNs, resulting in higher computational expense but superior capability in handling complex sequence data. BERT uses a different approach from RNNs, LSTMs, and gated recurrent units; it is a pretrained language model that leverages a large text corpus and is constructed on a bidirectional transformer with a self-attention mechanism [25].

Owing to the complexity of tokenizing Korean, which has a word order and contains multiple morphemes in a single clause, the LSTM model uses the Kiwi tokenizer, which is suitable for generating a Korean corpus [26]. For the BERT model, its tokenizer was applied for pretraining with special tokens ([CLS] and [SEP]) to denote the beginning and end of a sentence.

In this study, the BERT model accessible through the Keras library was used, with all layers of the preexisting model being retrained. For the LSTM model, a single LSTM layer with 256 units was used to capture the long-term dependencies in the sequence data. The Softmax activation function was used in the final layer of both models to generate a probability distribution across each category for the final classification task. Training of the models spanned 50 epochs and incorporated an EarlyStopping callback to avoid overfitting. This callback was configured to halt training if no improvement in validation loss was noted across 15 consecutive epochs. The model configuration that achieved the minimum validation loss during training was preserved and used to make predictions on the test dataset and conduct subsequent evaluations.

#### In Context Learning Using GPT-4

As a novel approach for classifying the purpose of phone calls, this study leveraged the capabilities of GPT-4, extending beyond traditional deep learning models such as LSTM and BERT. Initially, five categories—Treatment, Records, Routine, Rescheduling, and Symptom—were used for classification. These categories were expanded to 9 by incorporating the chain of thought. This refined categorization approach was applied to the prompts used with GPT-4 to enable a more precise classification within context learning. For comprehensive reference, the original and refined prompts used across all models are thoroughly documented in Table S2 in Multimedia Appendix 1.

**Figure 2:**
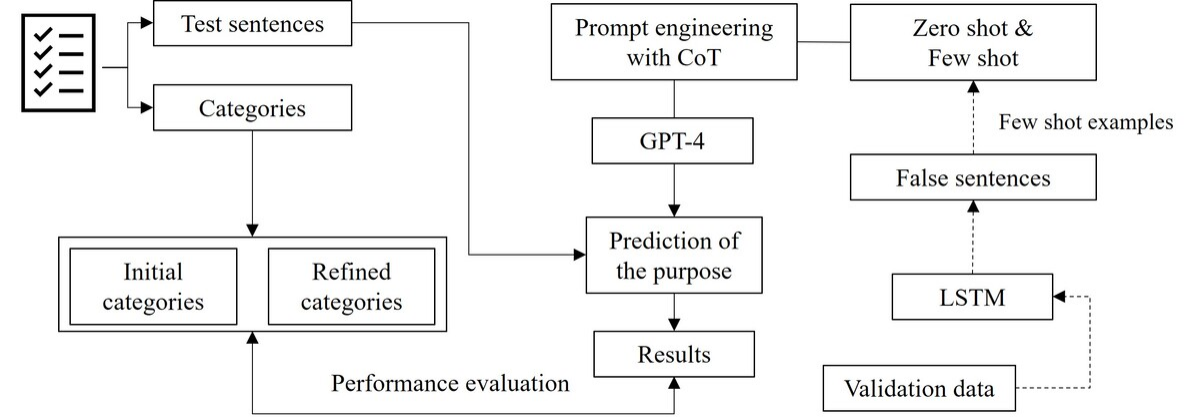
Classification process of initial and refined categories using GPT-4. CoT: chain of thought; LSTM: long short-term memory.

Figure 2 shows the use of GPT-4 for categorizing both the initial and refined categories. The categories were restructured using the test dataset, and the prediction outcomes were compared by applying GPT-4 without an example (zero-shot) and with a few examples (few-shot). In GPT-4 zero-shot, specific prompts for categories such as “Treatment,” “Records,” “Routines,” “Rescheduling,” and “Symptoms” were used to determine the purpose of the consultation. In contrast, the GPT-4 few-shot approach aims to improve the accuracy of the model by adding more examples of correct answers to the zero-shot approach. In this process, the LSTMs were used to predict the validation data, and example sentences were generated from sentences that were incorrectly predicted. Example sentences are presented in Table S3 in Multimedia Appendix 1. Various settings, including temperature and role assignments in the system prompt, were experimented with in an attempt to achieve more consistent responses from GPT-4. However, no notable improvements or distinctive patterns were observed as a result of these adjustments. Consequently, GPT-4’s default web interface settings were applied for all experiments in this study.

#### Performance Evaluation Methods

For the evaluation, 1000 randomly selected sentences from the 2020 dataset were used as the evaluation data. Each sentence was assigned both the existing labels and new labels reviewed by professional nurses. While 9 categories were used in the prompt construction, accuracy and recall scores were analyzed for 5 categories to evaluate the model’s performance. This was because the goal was to assess the model’s performance and accuracy based on categories commonly used in the actual hospital call center.

### Ethical Considerations

The study was approved by the institutional review board of the Samsung Medical Center (SMC 2021-10-001). Our research uses retrospective data containing anonymized personal information and has received a waiver of informed consent from the subjects.

## Results

### Classification Results With Initial Categories

**Figure 3:**
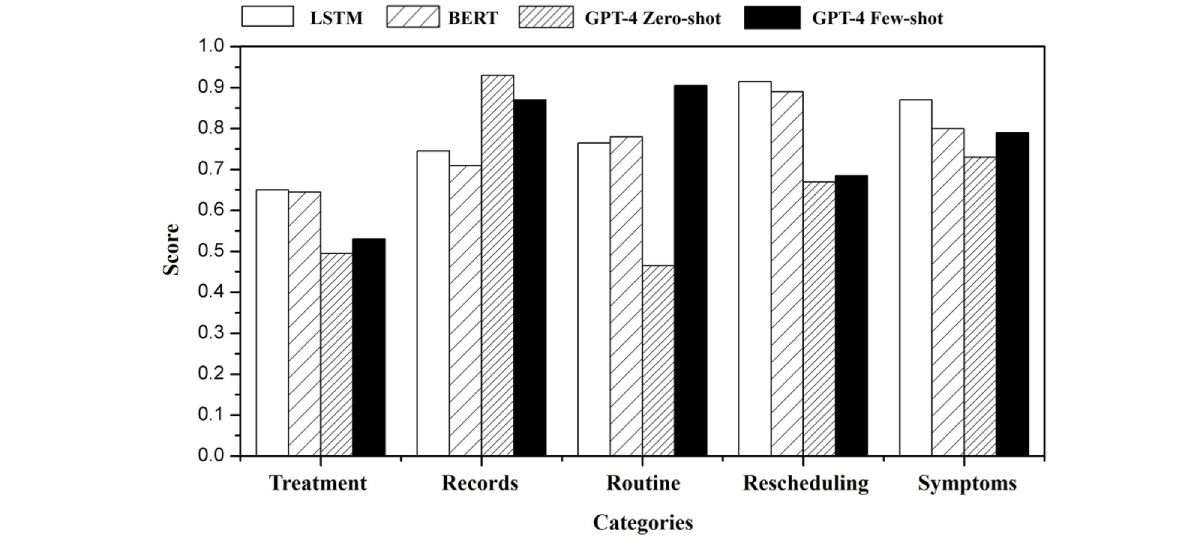
Comparison of long short-term memory (LSTM), bidirectional encoder representations from transformers (BERT), and GPT-4 in consultation call purpose classification with initial categories.

Figure 3 compares the prediction results of LSTM, BERT, GPT-4 zero-shot, and GPT-4 few-shot approaches based on the initial categories. According to these preliminary results, LSTM and BERT outperformed GPT-4 in most categories. In the “Treatment” category, all models showed relatively low performance, while GPT-4 slightly outperformed the others in the “Records” category. In the “Routine” category, GPT-4 few-shot achieved the highest accuracy; however, LSTM showed the best performance in the “Rescheduling” and “Symptoms” categories.

However, these results should be interpreted with caution. The differences may be due to uncertainty in the initial category definitions rather than variations in model capability. The boundaries between categories are often ambiguous and overlapping, potentially distorting the evaluation of model performance.

Thus, these preliminary results suggest that the need lies not only in comparing model performance but also in reviewing and improving the category definitions.

### Confusion Matrix Results Between Initial and Refined Categories

**Figure 4:**
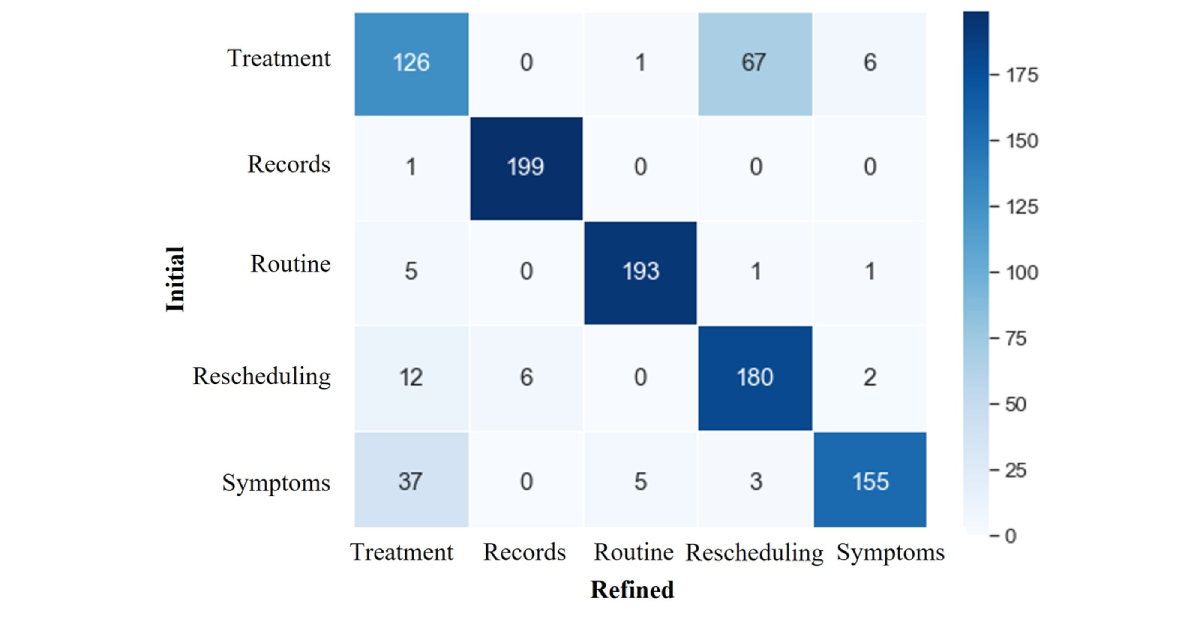
Comparison of confusion matrix results between initial and refined categories.

To address the uncertainties present within the test dataset, the predefined categories were thoroughly reassessed by multiple researchers. Figure 4 shows confusion matrices, a standard metric for evaluating the efficacy of classification models that show the differences between the original and expert-refined categories.

In the “Treatment” category, approximately 63% of the sentences matched correctly, but 33.5% were incorrectly classified as rescheduling and 3% as symptoms, indicating a significant overlap and misunderstanding among these categories. For the “Rescheduling” category, a precise correlation with the intended classification was evident in 90% of the sentences; however, approximately 6% were misclassified as treatment. The “Symptom” category had 77.5% correct matches, but 18.5% were confused about the treatment. In contrast, the “Records” and “Routine” categories demonstrated exceptional precision with accuracies of 99.5% and 96.5%, respectively, indicating a well-defined lexical and semantic distinction within these categories, which is conducive to accurate classification. This analysis highlighted considerable ambiguity, primarily among the “Treatment,” “Symptoms,” and “Rescheduling” categories, leading to a strategic refinement of the classification prompts to resolve these issues.

### Classification Results With Refined Categories

**Figure 5:**
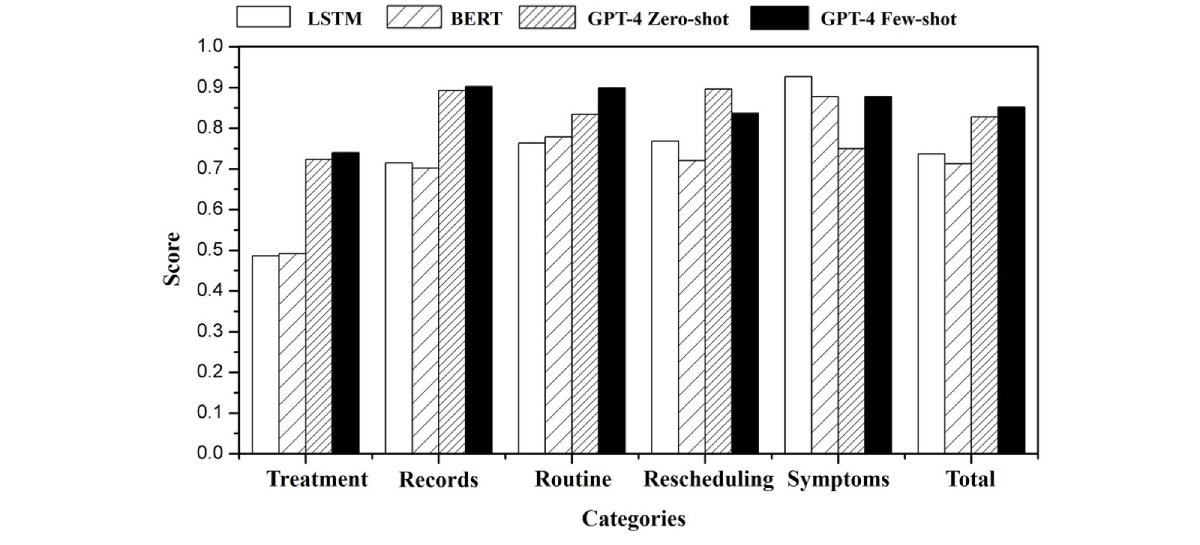
Performance metrics of long short-term memory (LSTM), bidirectional encoder representations from transformers (BERT), and GPT-4 models in consultation call purpose classification with refined categories.

When constructing the GPT-4 prompts, the performance improved when the 5 categories were further subdivided based on the chain of thought. Figure 5 and Table 1 display the results evaluated using the recall score for each category as well as the overall accuracy. The GPT-4 few-shot model outperformed the LSTM and zero-shot models in most categories, achieving the highest recall for records at 0.9024, routine at 0.8995, and symptoms at 0.878. The zero-shot model exhibits the best performance in the “Rescheduling” and “Treatment” categories, with recall scores of 0.8964 and 0.7238, respectively. The LSTM model obtained the highest recall in the “Symptoms” category at 0.9268, but had the lowest performance in all other categories. Considering the total accuracy, the GPT-4 few-shot model achieved the highest score of 0.852, followed by the zero-shot model at 0.828 and the LSTM model at 0.737.

**Table 1:** Performance comparison of GPT-4 zero-shot and few-shot on refined categories.

				Discriminative models					GPT-4 (with CoT^a^)			
				LSTM^b^		BERT^c^		Zero-shot				Few-shot
**Recall score**												
	Treatment			0.486		0.492				0.724		0.740
	Records			0.715		0.702				0.893		0.902
	Routine			0.764		0.779				0.834		0.899
	Rescheduling			0.769		0.721				0.896		0.837
	Symptoms			0.927		0.878				0.750		0.878
**Total**												
		Accuracy	0.737		0.713		0.828				0.852	

^a^CoT: chain of thought.

^b^LSTM: long short-term memory.

^c^BERT: bidirectional encoder representations from transformers.

## Discussion

### Principal Results

The findings of this study highlight the potential challenges of using deep learning models, specifically LSTM and BERT, as well as the novel application of GPT-4 for categorizing phone calls in a medical context. Analyzing a dataset that includes transcripts from consultations of a patient with cancer has provided valuable perspectives on the efficiency of various learning approaches and the influence that the way categories are defined can have on classification accuracy.

In particular, GPT-4 few-shot exhibited an impressive capacity to use a limited set of specific examples, leading to notable enhancements in classification efficacy compared with discriminative models. With refined categories, few-shot GPT-4 achieved an accuracy of 85.2%, outperforming LSTM (73.7%) and BERT (71.3%). These results demonstrate that GPT-4 few-shot is effective in overcoming issues arising from ambiguous category boundaries within datasets characterized by complex and subjective features, which are common in telephone consultations with patients with cancer. This is comparable to other LLM studies that reported an accuracy of more than 90% in organizing structured data from medical records [27,28].

The evaluation of GPT-4’s predictive capabilities yielded encouraging results but encountered difficulties. Notably, errors in classification were observed, specifically within medical documentation contexts, such as the removal of surgical adhesives, administration of topical medications, and symptom consultations related to medication and treatment references. To delve deeper into these findings, GPT-4 few-shots were used to predict multiple categories. Subsequently, a comparative analysis was performed between the predicted outcomes and categories revised by the experts. The examination revealed 55 instances of misclassification, representing a performance enhancement of more than 10% compared with single-category predictions.

Table 2 shows the representative sentences among the misclassified sentences. Sentences related to medical documentation, particularly those concerning prescription issuance, were predominantly misidentified as inquiries related to medical appointments. Moreover, questions about the removal of surgical tape or the application of ointment were incorrectly classified as related to daily living activities rather than consultations on symptoms or surgical and medical procedures. Certain sentences were misclassified as consultations about symptoms due to mentions of drugs and treatments rather than being recognized as related to routine daily activities. However, we need to consider the prevailing assumption that there is often a discrepancy between the patient’s surface questions and the actual problems that physicians must address [29,30]. This is because doctors and nurses refer not only to patients’ comments but also to medical records to determine patients’ actual needs. Therefore, it may be difficult for LLMs to make in-depth inferences based solely on the patient’s comments.

**Table 2:** Example of a sentence that was inaccurately predicted when compared with multicategories.

Sentences	Initial categories	Multicategories
The patient inquires if they can remove the tape applied after the prosthetic surgery on their own and how to apply the ointment. Please confirm. 환자, 보형물 수술후 테잎 붙여진것을 임의로 떼도 될지와 연고 바르는 것은 어떻게 하는지 상담 원하십니다. 확인부탁드립니다.	Routine	Treatment
TF2 general patient GS is currently receiving treatment from the Department of Breast Surgery and wishes to get a prescription—directed to the Gastroenterology Center to inquire about making an appointment for tomorrow; no issues related to COVID-19. TF2 일반환자GS 유방외과 진료보고 있는데 처방전을 발급 받을려고함-> 소화기 센터로 안내해줘서 내일 진료예약할수 있는지 문의-코로나 관련 이상없음	Records	Treatment, Rescheduling
Professor’s patient, currently undergoing chemotherapy, reports discomfort in the prostate area, and is taking supplements. They are inquiring if it is safe to take the supplements along with chemotherapy. Please advise. 교수님환자, 항암제 복용중인데 전립선쪽이 안좋은셔서 영양제 복용중이라고 합니다. 항암제랑 영양제 함께 복용가능한지 문의합니다 상담부탁드립니다..	Routine	Treatment, Symptom
The patient has been taking tamoxifen and experienced menstruation during the month of surgery but has not had any periods since then, until they resumed on February 22. Please confirm if it would be appropriate to schedule an appointment directly with the gynecology department, or if the patient should attend another outpatient consultation first. 타목시펜 복용중으로 수술하신 달에는 생리하셨고 그이후로는 생리를 안하시다가 2/22일부터 생리를 하신다고 합니다. 바로 산부인과쪽으로 예약을 해드려도 될지, 아니면 외래진료를 한번 더 받으셔야 할지 확인부탁드립니다.	Symptom	Treatment, Rescheduling
The daughter mentioned that she had inquired about the possibility of hospitalization after speaking with the nurse at IM6 (hemato-oncology) and was told that they would consult and call back. She now wishes to be admitted today and requests to speak again with the nurse involved in the previous consultation. Thank you. 딸아까 im6 (혈액종양내과) 간호사와 통화후 입원여부 여쭤봤을때 상의하고 다시 전화드리겠다고 했었다고 합니다. 오늘 입원하겠다며 해당 상담했던 간호사와 재통화원하십니다. 감사합니다.	Treatment	Rescheduling Records

### Limitations

This study had several limitations that require further methodological consideration. The best performance was observed when prompts were constructed based on more detailed categories derived from the initial categories, suggesting that the results may not be generalizable. For the LSTM model, we used the Kiwi tokenizer, a tool specifically designed for Korean, which differs from tools for other languages in its handling of syntactic properties and morpheme normalization. This difference can impact the generalizability of the model performance across different languages. Furthermore, the data used in this study were not directly sourced from the content provided by the patients but rather based on the information summarized by the consultants. However, consultants (or counselors) are typically trained to write the exact words spoken by patients verbatim. Consequently, there are clear limitations for other researchers in obtaining the same level of performance as the results of this study.

### Comparison With Prior Work

Despite encountering challenges in inferring the contextual purpose of calls, this study significantly contributes to broadening the application of LLMs in the field of oncology. Currently, LLMs play versatile roles in clinical settings, such as extracting meaningful outcomes from medical records or supporting clinical decision-making related to treatment options [27,28,31,32]. Some studies have attempted to use LLM to support clinician workflows in emergency department triage [33,34]. Our study suggests that LLMs can help both clinical and nonclinical workers develop a standardized perspective of problem identification in hospital call centers for patients with cancer. Although the accuracy and repeatability of LLMs may be controversial depending on the tasks performed by them, our study also suggests that LLMs are an efficient tool for using enormous and varied real-world data in the clinical environment [35,36].

### Conclusions

This study demonstrated the potential of leveraging LLMs, particularly GPT-4, to understand the complex and diverse intentions of patients with cancer during telephone consultations. The superior performance of GPT-4 over retrained discriminative models highlights its ability to consistently identify patient intent without laborious annotation. However, challenges in classification accuracy underscore the importance of carefully defining categories and refining prompts to fully harness the power of LLMs. As more advanced LLMs emerge, exciting opportunities will emerge to further enhance the efficiency and standardization of telephone consultations for better cancer care. In the future, further study should be conducted to confirm whether an LLM-based telephone consultation system can enhance the work productivity and efficiency of hospital staff in identifying the unmet needs of patients and providing appropriate support.

## Appendix

Multimedia Appendix 1 GPT-4 prompt applied in this study.

## Abbreviations

LLM: large language model
BERT: bidirectional encoder representations from transformers
LSTM: long short-term memory
RNN: recurrent neural network

## Data Availability

Data supporting the findings of this study are available upon request from the corresponding authors. The codes supporting the findings of this study are available upon request from the corresponding authors.
