# Supplementary material for: Leveraging Large Language Models for Improved Understanding of Communications With Patients With Cancer in a Call Center Setting: Proof-of-Concept Study
Source: J Med Internet Res. 2024 Dec 11;26:e63892. doi: 10.2196/63892 (PMC11669882; doi:10.2196/63892)
Supplement: Multimedia Appendix 1 [file jmir_v26i1e63892_app1.docx]

Table S1. Categories and definitions of consultation purposes.

| 5 - Categories | 9 - Categories | Detail definitions |
| --- | --- | --- |
| Treatment | - Diagnostic and medical treatment | - Explanation of prescribed tests, Guidance on retest procedures, Detailed explanation of test results |
|  | - Surgery & Procedure Consultation | - Explanation of treatment methods (chemotherapy, radiation, surgery, etc.), Requests for dental professional verification |
|  | - medication and treatment management | - Compatibility of traditional medicine prescriptions, Decisions on pausing, re-prescribing, or continuing medication due to symptoms |
| Records | - Medical services and management | - Inquiries about medical record details, requests for medical records issuance, requests for amendments in recorded details |
| Routine | - Vaccination Consultation | - Primary prevention (vaccinations, flu shots, dental cleaning, etc.) |
|  | - Lifestyle Consultation | - Aesthetics (Botox, plastic surgery, perms, dyeing, etc.), Travel (domestic, international), Leisure & Exercise (swimming, hiking, weight training, personal training, etc.), Food intake (supplements, juices, herbal medicine, specific foods, etc.) |
| Rescheduling | - Appointment requests | - Purpose of scheduling medical appointments or surgeries |
|  | - Schedule changes | - Requests to change or cancel scheduled treatments or surgeries |
| Symptom | - Disease & Symptom Consultation | - Consultation on symptoms before, during, or after treatment, coping strategies for experienced symptoms, inquiries on the relation between symptoms and treatment |

Table S2. Adapting zero-shot and few-shot learning to classify telephone consultations.

| Prompt for Zero Shot (without CoT) | | |
| --- | --- | --- |
| Categories | Inclusion Criteria | |
| - Treatment | - Requests for explanation about treatment methods (chemotherapy, radiation, surgery, etc.), inquiry about the compatibility of prescribed herbal medicines from oriental clinics, requests for verification of dental medical staff, decisions on drug holiday, re-prescription, and continuation of drugs prescribed due to symptoms, requests for explanation and re-prescription of prescription content, verification requests from medical staff regarding patient's actions based on the results of opinion letters, requests for guidance on examination methods, detailed explanation requests regarding test results, inquiries about the mechanism of drugs, inquiries about drug combination due to comorbid diseases, verification requests from medical staff regarding prescribed drugs and procedures for treatment and symptom relief, inquiries about hospitalization schedules related to surgery/procedure/tests. | |
| - Medical Records | - Inquiries related to entries in medical records, requests for issuance of medical records, requests for modifications of recorded items. | |
| - Daily Life | - Beauty (Botox, plastic surgery, perm, dyeing, etc.), travel (domestic, international, etc.), leisure and exercise (swimming, hiking, weightlifting, personal training, etc.), food intake (supplements, juices, herbal ingredients, specific foods, etc.), dental treatment (root canal, cavity treatment, etc.), primary prevention (vaccinations, scaling, etc.). | |
| - Symptom Consultation | - Complaints about symptoms occurring before, during, or after treatment, inquiries on how to deal with experienced symptoms, inquiries about the relationship between experienced symptoms and treatment, consultations on side effects due to treatments or drugs, sentences where symptoms are directly mentioned. | |
| - Schedule Changes | - Sentences that directly mention requests for changes or cancellations of appointments, tests, surgeries, etc. Inquiries/consultations about schedules/durations are not related to schedule changes. | |
| - Main prompt to predict the purpose of the call | | |
| - Please consider the purpose of the call in the sentences provided and respond with the appropriate category. The example response format should be "Sentence Number_Category". | | |
| Prompt for Detailed Categories (with CoT) | | |
| Categories | Detailed Categories | Inclusion Criteria |
| - Treatment | - Testing and Consultation | - Decisions regarding the necessity of tests, consultations on specific types of tests (ultrasound, MRI, CT, etc.), interpretation of test results and subsequent actions, consultations directly related to testing procedures, discussions based on test results regarding medical direction or treatment plans. Clarify that requests for medical document issuance fall under "Medical Services and Management.". |
|  | - Medication and Treatment Management | - Inquiries about combining medications prescribed by traditional Korean medicine, requests for confirmation from dental professionals, decisions regarding pausing, re-prescribing, or continuing medication already prescribed for symptoms, questions about the mechanism of action of medications, inquiries about combining medications due to comorbid conditions, requests for medical confirmation regarding prescribed medications or treatments for symptom relief or treatment. |
|  | - Surgery and Procedure Consultation | - Eligibility for surgery, pre- and post-surgery care, scheduling for surgery, duration and likelihood of hospitalization, guidelines for medication intake related to surgery |
| - Medical Records | - Medical Services and Management | - Management of medical records, requests for amendments related to medical records, requests for medical documents (medical records, diagnosis letters, opinions, surgical confirmations, exceptional medical expenses), requests for medical consultations for documentation purposes, administrative and coordination-related inquiries regarding the provision of medical services. |
| - Daily Life | - Vaccination Consultation | - Primary prevention (vaccinations, flu shots, scaling, etc.). |
|  | - Daily Life Consultation | - Cosmetics (Botox, plastic surgery, perms, dyeing, etc.), travel (domestic, international, etc.), leisure and exercise (swimming, hiking, weight training, personal training, etc.), food intake (supplements, juices, herbal medicines, specific foods, etc.) |
| - Symptom Consultation | - Symptom Consultation | - Complaints of symptoms before, during, or after treatment, inquiries on coping with experienced symptoms, questions on the relation between experienced symptoms and treatment, consultations on side effects due to treatments or medications, sentences directly mentioning symptoms. |
| - Schedule Changes | - Schedule Changes | - Direct mentions of requests for changes or cancellations of appointments for medical examinations, surgeries, etc. Inquiries about dates/durations do not relate to schedule changes. |
|  | - Appointment Requests | - Requests for scheduling or consulting on surgeries, procedures, hospital admissions, appointments for medical examinations, or tests. |

Table S3. Example sentences applied for few-shot learning for each category.

| Categories | Detailed categories | Examples |
| --- | --- | --- |
| - Treatment | - Testing and Consultation | - We were supposed to have blood drawn before Professor’s 11:20 consultation, but it was forgotten. They're coming from Gyeongnam and said they will arrive by 11:20. Please check what should be done about the blood draw." - "Consulted with the department regarding the patient of professor before their consultation and advised to get tested at a nearby hospital. They said they have questions about the test items and strongly request department consultation. Please consult. |
|  | - Medication and Treatment Management | - "Patient of Professor inquiring if they can continue taking their prescribed hormone medication during the blood test scheduled for 9.27." |
|  | - Surgery and Procedure Consultation | - "Patient of Dr. said they are scheduled for a procedure. They want to change the method of the procedure and requested to speak with the person in charge. I've left a message as the connection was difficult. Please assist." |
| - Medical Records | - Medical Services and Management | - "Patient of Professor says there was an error in issuing their disability diagnosis letter and requests reissuance at the earliest possible date. Please check the schedule." |
| - Daily Life | - Vaccination Consultation | - "Please check if the flu vaccination is available. Thank you, take care." - "Patient of Dr. was admitted on 1/21 and has surgery on 12/2. They were informed that vaccination is not possible before surgery, and now they wish to consult on when they can get the pneumonia vaccine after the surgery." |
|  | - Daily Life Consultation |  |
| - Symptom Consultation | - Symptom Consultation | - "The patient themselves called ==> The guardian noticed 2~3 days ago. No pain or foreign body sensation. Advised to seek consultation if discomfort arises." - "Patient who consulted with Dr. continues to experience hematuria post-surgery and wishes to consult on the symptom.". |
| - Schedule Changes | - Schedule Changes | - "Patient of Dr. wishes to cancel the cystoscopy test on 1/19 due to personal reasons. No need to call back, thank you." - "The patient has been feeling unwell recently and wishes to have their test rescheduled to the earliest possible date. Advised to see a doctor first, but they requested a test change, asking if it wouldn't confirm their condition.". |
|  | - Appointment Requests |  |
